# A preliminary study of dyads of stroke patients and their female partners: Exploring the role of spirituality, religiousness and quality of life in rehabilitation

**DOI:** 10.1192/j.eurpsy.2021.1137

**Published:** 2021-08-13

**Authors:** V. Giannouli, K. Giannoulis

**Affiliations:** 1 Institute Of Neurobiology, Bulgarian Academy of Sciences, Sofia, Bulgaria; 2 Faculty Of Theology, Aristotle University of Thessaloniki, Thessaloniki, Greece

**Keywords:** Spirituality, religiousness, quality of life, stroke

## Abstract

**Introduction:**

The concepts of spirituality and religiousness has not been investigated so far in patients after stroke.

**Objectives:**

The aim of this study is to explore whether self-reports in two questionnaires measuring the personal experience of spirituality and religiousness can influence quality of life and estimations of rehabilitation in male older adult patients and their wives when compared with control dyads.

**Methods:**

Fifteen male stroke patients and their wives participated one year after their hospitalization for stroke. The mean age of the patients and their wives was 75.58 years (SD = 7.50, range 61-90), level of education 15.47 years (SD = 3.82). In addition to that, fifteen married couples with similar demographics, were also measured. Depressive symptoms of the participants were assessed with the 15-item Geriatric Depression Scale. Family dyads consisting of an older adult and one family member, in all cases the wife, also responded to the Daily Spiritual Experience Scale, the Systems of Belief Inventory (SBI-15R), and the Satisfaction with Life Scale (SWLS). A 5-point Likert scale question was also administered examining the opinion of rehabilitation achieved.

**Results:**

indicated that there was a statistically significant difference between the two groups regarding the levels of spirituality, religiousness and quality of life in both partners, with the stroke patient dyad showing lower scores, but positive stronger correlations.

**Conclusions:**

Although spirituality, religiousness and quality of life are lower in the stroke patient dyad, they show significantly statistical positive correlations in older adults suffering from stroke as well as their wives.

